# Increasing the Power of Polyphenols through Nanoencapsulation for Adjuvant Therapy against Cardiovascular Diseases

**DOI:** 10.3390/molecules26154621

**Published:** 2021-07-30

**Authors:** Lucileno Rodrigues Trindade, Davi Vieira Teixeira da Silva, Diego dos Santos Baião, Vania Margaret Flosi Paschoalin

**Affiliations:** Chemistry Institute, Federal University of Rio de Janeiro, Avenida Athos da Silveira Ramos 149, Cidade Universitária, Rio de Janeiro 21941-909, Brazil; lucileno.trindade@gmail.com (L.R.T.); davivieira@ufrj.br (D.V.T.d.S.); diegobaiao20@ufrj.br (D.d.S.B.)

**Keywords:** nanocarriers, antioxidants, bioavailability, cardiovascular health, polyphenol-loaded nanoparticles, pre-clinical trials

## Abstract

Polyphenols play a therapeutic role in vascular diseases, acting in inherent illness-associate conditions such as inflammation, diabetes, dyslipidemia, hypertension, and oxidative stress, as demonstrated by clinical trials and epidemiological surveys. The main polyphenol cardioprotective mechanisms rely on increased nitric oxide, decreased asymmetric dimethylarginine levels, upregulation of genes encoding antioxidant enzymes via the Nrf2-ARE pathway and anti-inflammatory action through the redox-sensitive transcription factor NF-κB and PPAR-γ receptor. However, poor polyphenol bioavailability and extensive metabolization restrict their applicability. Polyphenols carried by nanoparticles circumvent these limitations providing controlled release and better solubility, chemical protection, and target achievement. Nano-encapsulate polyphenols loaded in food grade polymers and lipids appear to be safe, gaining resistance in the enteric route for intestinal absorption, in which the mucoadhesiveness ensures their increased uptake, achieving high systemic levels in non-metabolized forms. Nano-capsules confer a gradual release to these compounds, as well as longer half-lives and cell and whole organism permanence, reinforcing their effectiveness, as demonstrated in pre-clinical trials, enabling their application as an adjuvant therapy against cardiovascular diseases. Polyphenol entrapment in nanoparticles should be encouraged in nutraceutical manufacturing for the fortification of foods and beverages. This study discusses pre-clinical trials evaluating how nano-encapsulate polyphenols following oral administration can aid in cardiovascular performance.

## 1. Introduction

Natural bioactive agents are attractive molecules, as their implicit safe status allows them to be tested as specific or multiple target regulators in signaling or functional human pathways, while also comprising promising therapeutic adjuvant candidates against multifactorial and complex physiopathological conditions, including degenerative diseases and metabolic dysfunctions. In food matrices, natural bioactive compounds contribute to color [1], bitterness, astringency [2], flavor [3], preservation, and safety [4]. Among the various naturally available bioactive molecules, polyphenols, which are secondary metabolites present in edible roots and plants, are associated with decreased risks concerning chronic, degenerative, and cardiovascular diseases when consumed regularly [5,6,7]. A large number of clinical trials have highlighted the therapeutic role of polyphenols on vascular disorders, playing a role in inherent illness-associate conditions such as inflammation, type 2 diabetes, dyslipidemia, hypertension, and oxidative stress [8,9,10,11]. Such findings have been compiled and reinforced in recent epidemiological data and meta-analysis [12,13,14,15].

**Figure 1:**
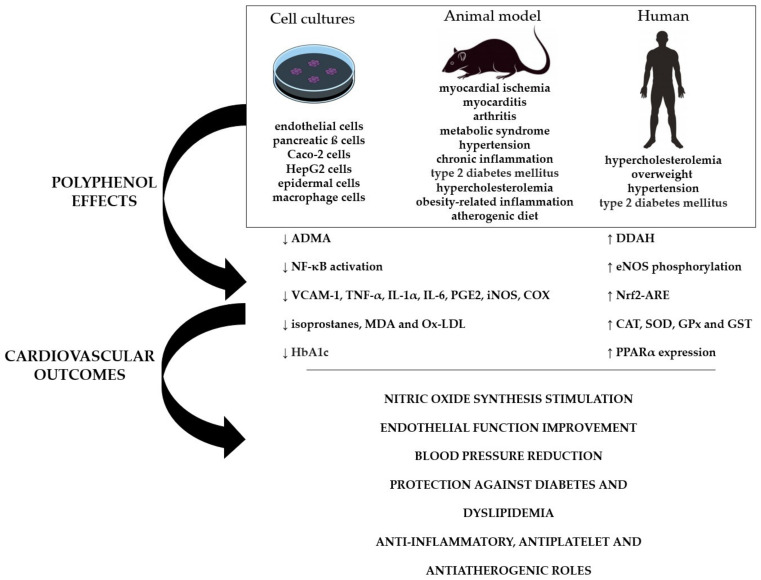
Protective role of polyphenols in different inflammatory and oxidative pathways in cells, animals, and humans that protect and improve the cardiovascular system.

Several mechanisms are involved in the cardioprotective role of polyphenols. These compounds increase nitric oxide (NO) release and vasodilation in endothelial cells through several nitric oxide synthase (eNOS) effects, including gene expression, catalysis activation by phosphorylation of the eNOS Ser1179 residue, and decreased asymmetric dimethylarginine (ADMA) levels, and an eNOS inhibitor, by increasing dimethylarginine dimethylamino hydrolase (DDAH) activity [16,17,18,19,20]. In addition, polyphenols positively regulate the expression of antioxidant enzymes by modulating the nuclear factor erythroid-2-related factor—antioxidant response element (Nrf2-ARE) [21]. Polyphenols also downregulate the inflammatory response, excessive reactive oxygen species (ROS) generation, and the activation of the redox-sensitive transcription factor nuclear kappa B (NF-κB), the master inflammatory response regulator [22]. Polyphenols also regulate lipid metabolism and diabetes, presumably through the expression and activation of the peroxisome proliferator-activated gamma receptor (PPAR-γ) [23,24,25,26]. Acting through these broad molecular mechanisms, polyphenols play a protective role against endothelial dysfunction and inflammation, resulting in cardiovascular protection (Figure 1).

Conversely, in vivo polyphenol performance may be limited due to the low hydrophilicity and poor intrinsic dissolution rate of these compounds or their physical or chemical instability [27,28]. In addition, these physicochemical characteristics may interfere in polyphenol bioavailability, as low absorption, scarce biodistribution, first-pass metabolism, poor penetration, and human organ accumulation should be expected [29,30,31].

**Figure 2:**
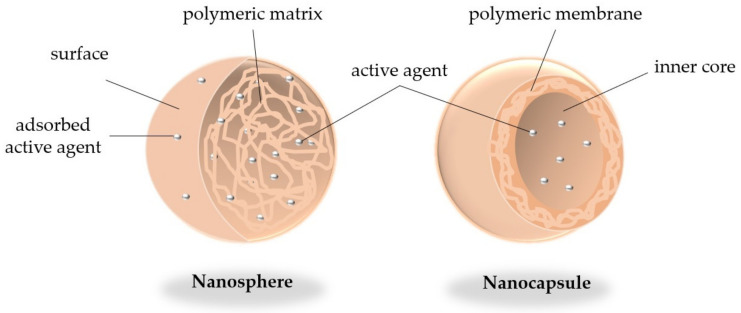
Nanospheres with the active agent adsorbed and dispersed on the polymeric surface (**left**); and nano-capsules with the active agent confined in the core surrounded by a polymeric membrane (**right**).

Nano-encapsulation is a submicroscopic technology used to carry active solid or liquid substances into a 10–1000 nm colloidal system, termed nanoparticles (NPs) [32]. NPs can be classified as nanospheres or nano-capsules, according to the distribution of their active principles. If their active principles are uniformly adsorbed onto the surface or bound to polymeric chains, they are called nanospheres, but if they are confined within a cavity or core surrounded by a polymeric membrane, they are termed nano-capsules. Both are created from a single polymer or a combination of distinct polymers, both natural or synthetic [33] (Figure 2).

Nano-encapsulation has been substantially applied in the formulation of novel drugs and as a high-technology food input in order to protect bioactive compounds such as polyphenols, essential oils, peptides, oligonucleotides, and antioxidants from oxidation or lysis by external factors, including pH, temperature variations, and exposure to light, ensuring their stability and increasing the half-lives of active principles. As a carrier system for therapeutic agents, nanoparticles can provide a controlled release of the active principle after oral, parenteral, or topical administration and, at the same time, guarantee better solubility and chemical structure stability, thus ensuring target achievement [33]. Due to their subcellular size similar to biomolecules, NPs are able to pass through the pores of the intestinal epithelium, cross biological barriers, including the blood-brain barrier, and penetrate tissues through microcapillaries, allowing for the delivery of therapeutic agents to specific organs and tissues and resulting in greater bioavailability [34,35,36,37,38,39,40]. It has been proposed that orally administered polyphenols are absorbed by transcellular diffusion in enterocytes and M cells and, to a lesser extent, by paracellular diffusion between cell junctions [41,42].

Foods can be enriched in polyphenols by adding NPs carrying pure and concentrated polyphenols obtained from edible plants or food grade microorganisms, such as yeast or acid lactic bacteria, in order to protect polyphenols from oxidative degradation and conferring all the aforementioned stability and advantages [43].

The main natural (or food-grade) polymers obtained from plants and vegetables used to nano-encapsulate polyphenols are presented herein. The ability of these polymers to preserve polyphenol structure, bioactivity, and gastrointestinal bioaccessibility are addressed. The current framework concerning possible polyphenol gastrointestinal toxicity, bioavailability, and bioactivity addressed through clinical trials is reviewed and, finally, the projection of the performance of nano-encapsulated polyphenols in aiding cardiovascular impairments is discussed.

## 2. Brief Overview of Nano-Encapsulated Polyphenols: Nature and Physiochemical Polymer Benefits

Natural polymers, such as carbohydrates, proteins and lipids, are the most abundant organic matter in nature and essential for the existence of living organisms, as they perform vital functions such as comprising structural cell and tissue components and acting in transport and catalysis processes as energetic reserves and in energy transformation, among others [44]. Due to these characteristics and their biodegradability, biocompatibility, and non-toxicity, natural polymers have been chosen as the wall material and vehicle for drug and nutraceutical delivery in micro- and nanodevices. As mentioned previously, the entrapment of these polymers in nanoparticles ensures greater physical-chemical polyphenol stability in the gastrointestinal tract concerning enzymatic activities and the gastrointestinal environment itself, as well as in the bloodstream.

### 2.1. Proteins

Proteins such as gelatin, albumin, and casein are the most used proteins to obtain NPs. Gelatin is an encapsulant agent obtained from the partial hydrolysis of collagen and has been used to nano-encapsulate polyphenols alone or associated with surfactants and polysaccharides with opposite charges, such as cellulose, carboxymethylcellulose, chitosan, or arabic gum [45,46,47,48].

Polyphenol adsorption onto gelatin NPs occurs through the formation of hydrogen linkages between phenolic rings and hydrophobic amino acids [49]. Gelatin nano-capsules loaded with catechins from Camellia sinensis were formed spontaneously by homogenization under magnetic stirring of the polyphenol-protein mixture. The NPs were 140.5 nm in size, with a zeta potential of 0.2 mV, and over 96% of the catechin was encapsulated using a formulation containing 2 mg·mL^−1^ of each compound. Hydrogen bonds between the phenolic and aliphatic hydroxyls of catechin and gelatin, respectively, allowed for the spontaneous formation of NPs without the use of surfactants and crosslinkers. The gelatin-catechin NPs retained their antioxidant activity as evaluated by 2,2′-azino-bis(3-ethylbenzothiazoline-6-sulfonic) acid (ABTS) and 2,2-diphenyl-1-picrylhydrazyl (DPPH) assays after 3 weeks stored at room temperature, indicating the effectiveness of gelatin, concerning the chemical and bioactive stability of catechin [50]. An enriched polyphenol extract obtained from cocoa was successfully encapsulated with a gelatin and surfactant mixture (2:2:2 *w*/*v* %) followed by the addition of glutaraldehyde (0.125:1, glutaraldehyde:gelatin ratio), resulting in NPs with a uniform morphology and diameters ranging from 130 to 200 nm [51]. In that study, an encapsulation efficiency of 77% was observed, comparing the phenolic extract with the nano-encapsulated form through a DPPH analysis. The encapsulation efficiency and the diameter of gelatin-NPs can vary depending on the method, encapsulated agent, polymer concentration, and combination with surfactants and other wall materials. These variables explain the discrepancies observed regarding the encapsulation percentages and diameters reported in several studies.

Karthikeyan et al. [52] prepared 294 nm gelatin-NPs, with a polydispersity index (PDI) of 0.29 and zeta potential of −18.6 mV, using glutaraldehyde as the crosslinking agent and span 80 to encapsulate 10 mg of resveratrol. The release of resveratrol during the first 12 h ranged from 28% to 40%, which can happen in a wide range from 1.4 to 10.5 pH. About 30% of resveratrol was quickly released, followed by a slower and sustained release up to 48 h achieving 63% to 80% of total resveratrol entrapped at alkaline pH. The behavior exhibited by gelatin NPs can be advantageous in oral administration, due to the possibility of quickly reaching an effective resveratrol concentration, protecting it for a prolonged timeframe under unfavorable pH conditions. Song et al. [53] prepared gelatin NPs loaded with the flavonoids genistein and icariin by the desolvation method under pH variations (2–5). The NPs were stable for 180 days at room temperature and uniform at pH 3.5, with the smallest diameter of 231 nm, (PDI of 0.104) and best zeta potential of +29.61 mV. The adsorption capacity of the flavonoids increased with increasing gelatin NPs concentrations, exceeding 90% when gelatin NPs concentrations were over 3 g·L^−1^ and at temperature and incubation time above 20 °C and 24 h, respectively. The adsorption capacity of icariin was significantly higher than genistein, possibly due to the greater number of hydroxyl (–OH) groups. The amounts of adsorbed genistein and icariin were greater than their individual adsorptions, most likely due to the presence of more hydrogen linkages between phenolic rings and hydrophobic amino acids [49]. This can be of paramount therapeutic importance due to the possibility of producing NPs composed of different polyphenol combinations with good physicochemical properties.

Other proteins used as polyphenol nano-encapsulant agents consist of human or bovine serum albumins, HSA and BSA, both presenting a molecular mass of 66.5 kDa, synthesized in the liver and representing the most abundant proteins in plasma [54]. Albumin participates in osmotic and colloidal plasma maintenance and in the transport of various endogenous compounds such as other proteins, cholesterol, and bile pigments through the bloodstream [55,56]. These proteins may act as nano-encapsulating polymers for nutritional supplementation and pharmaceutical purposes, as they improve the solubility of hydrophobic active agents and their circulation in the body, while allowing for better tissue targeting [57,58,59]. As an encapsulant, albumin exhibits chemical stability and the ability to form NPs without the use of other polymers or surfactants, although the latter contributes to forming smaller particles with low aggregation capacity [49,51].

BSA-NPs loaded with rutin were formulated by nano-spray drying by dissolving 5 mg of rutin and 25 mg of BSA in water (6.6 mL), ethanol (3 mL), and Tween-80 (0.4 mL). The NPs were 316 nm in size and only 32% of the rutin was encapsulated due to low affinity to BSA and thermal polyphenol degradation due to high spray drying temperatures (100 °C). Conversely, BSA-rutin NPs comprised a homogeneous and stable nano-capsule population (PDI 0.27) with a surface charge of −32.1 mV. Rutin entrapment guaranteed a time control release and increased rutin IC50 by two-fold compared with free rutin for 72 h, as determined by ABTS+ assays [60]. BSA-NPs containing quercetin were developed using the desolvation method, using 8% and 20% glutaraldehyde as the crosslinking agent, resulting in 152 and 132 nm NPs, respectively. Quercetin was efficiently encapsulated in both formulations (>85%), exhibiting a monodispersed distribution and physical stability, as verified by a low PDI value (<0.12) and high zeta potential (≈ −40 mV). Quercetin was released in a second order kinetic rate, where 6% was released in the first 30 min, followed by a slow 15% loss in the next 24 h and slower amount of 18% up to 96 h. It seems that NP-adsorbed quercetin was rapidly released, whereas diffusion within NPs required the relaxation of the polymeric structure, slowing down quercetin release. Nano-encapsulation protected the quercetin antioxidant activity, as revealed by the ABTS radical inhibition assay compared with its free form at pH 7.4. 

Thus, BSA-NPs maintain polyphenol molecule stability and allow for their controlled release, causing them to be gradually available to exert their physiological effects, while preserving their activity over time [61]. Acetaminophen, ipriflavone, daidzein, and genistein were successfully entrapped into BSA- and HSA-NPs, mostly through the desolvation method [62,63,64]. 

Curcumin nano-encapsulated in HSA 2% (*w*/*w*) and chloroform/water (1:19), freeze-dried, and stored for 3 months at room temperature displayed no morphological alteration concerning 130–150 nm nano-capsules formulated in chloroform/water (1:19). The prepared HSA-NPs enhanced curcumin solubility by 300-fold and displayed cryoprotectant properties [65].

Caseins, the major milk phosphoproteins, exhibit a micellar structure ranging from 50 to 300 nm, stabilized by hydrophobic interactions and bonds between calcium phosphate and serine residues, and have been applied in phenolic entrapment. Casein nanoparticles promote structural phenolic stability, enhancing their solubility and bioavailability [66,67,68,69]. Casein nano-capsules, sized 168.7 nm on average, were used to encapsulate curcumin, reaching up to 83% encapsulation efficiency by the spray drying method in a sodium caseinate and ethanol solution (40% *v*/*v*). Non-encapsulated curcumin displayed a 40-fold increased water dispersion, resulting in high antioxidant power as assayed by the Trolox assay (8.87 mM) when compared with the free form (<1 mM). The enhanced dispersion and uniform curcumin distribution in the reaction medium and surface area inherent to casein nano-capsules seem to facilitate the reaction kinetics and bioactivity of the active principle [70]. A similar protocol was successful applied in the entrapment of ferulic acid, resveratrol, and epigallocatechin in casein-NPs following spray-drying. The release of phenolics from casein-NPs seems to obey the Korsmeyer-Peppas kinetic model in a zero-order reaction by relaxation and erosion of the polymeric matrix, with no pH influence from 1.2 to 6.8 [71].

A novel approach in the production of stable NPs in the gastrointestinal environment has been developed using a liquid-liquid dispersion method applied in the encapsulation of ferulic acid in a zein-casein-lysine matrix. The NPs were 199 nm in diameter, displaying a slow and prolonged release of 4% of the polyphenol for 48 h at physiological pH (pH 7.4). The strong interaction between ferulic acid and the nano-capsule-matrix was a mandatory factor for its sustained release, maintaining polyphenol protection and, consequently, its biological effects for a long period of time. However, the lack of a kinetic profile determination at gastric pH comprises a study limitation regarding NP characterization and usage [72].

Finally, caseins were used to encapsulate the flavonoid epigallocatechin gallate (EGCG) alongside glucosamine (GA) at a 1:2:8 ratio (*w*/*w*/*w*) in order to promote greater chemical stability. The NPs were 186 nm in size, displaying a high encapsulation efficiency (86.8%) and zeta potential (−35.8 mV) and were monodispersed (PDI 0.10), indicating feasibility in encapsulating a mixture of polyphenols. The formulation preserved 90% and 80% of EGCG for 15 and 45 days at 4 °C, respectively, while totally degrading at 25 °C. When nano-encapsulation was followed by freeze-drying, 90% of EGCG remained stable for 12 months at 25 °C without any physicochemical changes [73].

### 2.2. Polysaccharides 

Polysaccharides have also been widely used to develop capsules, mainly for biomedical and pharmaceutical purposes, due to their availability, low cost, and ability to form NPs. Alginates, chitosan, and starch are the most applied as nanocarriers for the oral delivery of polyphenols [74,75,76].

Alginate is anionic and water-soluble, and its popular use in obtaining NPs resides in its gelation ability in the presence of di- and trivalent cations under mild formulation conditions [77,78,79,80]. The combination of sodium alginate and chitosan, another carbohydrate polymer, is frequent, as it allows for NP formation by electrostatic bonding between the negatively charged alginate carboxyl groups and the protonated chitosan amines. Nonencapsulated alginate-chitosan compounds exhibit greater thermal stability and a continuous and controlled release profile [81,82], and its application as a delivery vehicle for compounds with pharmacological properties favors cell uptake due to the mucoadhesiveness inherent to alginate and greater permeability provided by the association with chitosan [83,84,85]. Corroborating this, Das et al. [86] encapsulated curcumin through alginate and chitosan ion gelatinization, using pluronic F127 as a surfactant. NPs 100 nm in size exhibited a slow curcumin release, achieving 75% of the initial amount at 96 h. Furthermore, these NPs were also absorbed by cell cultures. Considering systemic uses, these properties can enhance NP uptake by target cells and tissues, due to the longer permanence of the polyphenol in internal compartments.

The possibility of enriching NPs for the delivery of a mixture of hydrophobic polyphenols was tested by Saralkar and Dash [87]. Curcumin and resveratrol were encapsulated together in alginate-calcium chloride (CaCl_2_) particles through gelatinization and emulsification with Tween-80, forming 57 nm NPs. Resveratrol was efficiently encapsulated compared to curcumin (70.9 vs. 49.3%), while curcumin exhibited a slower release rate throughout 24 h (16.3 vs. 87%) and was better captured by cells compared resveratrol, demonstrating that alginate is a potential nanocarrier for hydrophobic polyphenols.

In an optimized methodological approach, quercetin was encapsulated by chitosan gelatinization using sodium tripolyphosphate (TPP) as a crosslinker, followed by polyelectrolytic complexation with sodium alginate using alginate-chitosan at a 1:2 ratio and quercetin at 7.5 mg·mL^−1^ [88]. NPs ranged from 118 to 255 nm and high encapsulation efficiency (82.4%), and quercetin was released in a biphasic pattern, reaching 78% in 8 h with the rest releasing gradually for up to 24 h. In this formulation, alginate promoted chitosan stability at an acid pH (5.5–6.5), its soluble range, and prevented the early exposure of quercetin to the gastric environment and consequent enzymatic degradation.

Starch has been applied as a wall material in nanotechnology, due to its solubility, emulsification, and biocompatibility characteristics. Its versatility relies on its structural-derivatives, maltodextrin and cyclodextrin, obtained by physical and chemical methods [89,90]. The amylose and amylopectin ratio, as well as its branching pattern and molecular weights, can be managed to influence NP size and its ability to encapsulate and retain active agents [91,92,93]. As a polyphenol carrier, starch can improve bioaccessibility in the gastrointestinal fluid and maintain antioxidant capacity in addition to providing stability against harsh conditions such as heat, radiation, and hyperosmotic environments [75]. Starch-NPs (1% *w*/*v*) loaded with curcumin (0.25 mM) by nano-precipitation using ethanol, an oil mixture (cyclohexane, sunflower oil, and oleic acid), and a surfactant, formed 87 nm NPs with 78% loading efficiency. Due to high starch hydrophilicity, greater surface area, and reduced size, NPs exhibited greater interaction with aqueous media and resulted in higher curcumin solubility compared to its free form. In addition, the use of starch NPs allowed for a controlled curcumin release of approximately 93% for 10 days under physiological pH [94].

The loading capacity of starch depends on amylopectin content. Starch obtained from different edible vegetables such as pea (*Pisum sativum*), corn (*Zea mays*), and potato (*Solanum tuberosum*), have been applied in quercetin encapsulation by the nanoprecipitation method using 0.1 M HCL as the non-solvent. The higher the amylopectin content, the better the quercetin entrapment and antioxidant activity, as follows: potato (49% loading) > pea (44% loading) > corn (20% loading) [95].

Other starch sources, such as water chestnut (*Eleocharis dulcis*), horse chestnut (*Aesculus hippocastanum*), and lotus stem (*Nelumbo nucifera*), have been used to encapsulate resveratrol, producing 691, 419, and 797 nm NPs, respectively, with an average encapsulation efficiency of 79%. Nano-encapsulation provided thermal stability to resveratrol, observed by the increases in NP transition temperatures when exposed to 200 °C [96].

In addition to the aforementioned assays, starch has also been applied for the nano-encapsulation of catechin, epicatechin, EGCG, and proanthocyanidin nano-encapsulation, and the resulting NPs exhibited chemical stability under different conditions, confirming the viability of starch in polyphenol delivery [75].

Chitosan is widely applied for nano-encapsulation due to its physical-chemical characteristics, including the ability to form gels by ionic bonds and its cationic nature, conferring mucoadhesiveness through electrostatic interactions and favoring the interaction of the transported agent with the mucus layer of epithelial surfaces [97,98,99,100].

Chitosan-NPs are generally prepared by gelation with TPP, a crosslinking polyanion widely applied due to its non-toxicity, multivalence, and the ability to form a gel, as mentioned previously [101,102,103]. Ionic interactions also occur inside NPs between the chitosan-TPP matrix and the encapsulated polyphenol, through the hydrogen bonds of the OH groups [104,105]. Such interactions promote a slow (approx. 100 h) and continuous NP release in addition to retaining antioxidant capacity and ensuring stability to prolonged exposure at high temperatures [105]. Chitosan is also used to deliver natural antibiotics, antioxidants, proteins, and dyes [106,107,108].

Recently, chitosan (1 mg·mL^−1^) cross-linked with TPP (0.5 mg·mL^−1^), was used to encapsulate quercetin and myricetin (5 mg·mL^−1^), generating 123.75 nm and 153 nm NPs, respectively. Both NPs exhibited high retention capacity greater in myricetin (89% vs. 82%) due to the presence of an extra hydroxyl in ring B, allowing for greater interaction with the positively charged matrix. The chitosan-TPP NPs provided a controlled polyphenol release at physiological pH (7.4) during 96 h, contributing to their prolonged antioxidant effect in relation to free flavonoids [109].

Through a single-factor optimization algorithm, carboxymethyl chitosan (CMC) cross-linked with CaCl_2_ was used to encapsulate resveratrol. NPs sized 155 nm and exhibiting 44% encapsulation efficiency and a zeta potential of −10.3 mV were obtained using CMC and CaCl_2_ at 5 mg·mL^−1^, 10:1 water/chloroform and 1:1000 water/surfactant. The antioxidant activity of resveratrol was substantially maintained in the formulated NPs compared with free resveratrol [110].

Similarly, He et al. [111] applied the response surface methodology mathematical model to establish anthocyanin, CMC, and chitosan chloridate (CHC) concentrations for the synthesis of stable NPs in beverages, with maximum anthocyanin encapsulation efficiency and minimum particle size. CMC, CHC, and anthocyanins were found to be optimal for the synthesis of NPs at 2.86, 0.98, and 5.97 mg·mL^−1^, respectively, resulting in 219 nm sized NPs and 63% encapsulation. The anthocyanin-NPs were highly stable beverages, maintaining 84% encapsulation for 35 days at 4 °C, 68.4% for 12 days at 25 °C, and 30% for 9 days at 40 °C. However, free anthocyanins were less stable, at 71.2%, 49.7%, and 6.3% under the same conditions [111].

Other polyphenols, such as gallic acid, catechin, procyanidins, ferulic acid, quercetin, and polyphenols from green tea extract (*Camellia sinensis*) have also been tested as active agents in chitosan NPs [112,113,114,115,116,117]. In short, chitosan NPs are able to effectively encapsulate polyphenols, providing thermal stability, stability in suspensions, solubility, and sustained release, while still retaining antioxidant polyphenol properties. Therefore, chitosan-NPs represent a promising vehicle for polyphenol delivery as they fulfill the criteria for safe nano-encapsulating natural matrices, such as biocompatibility and non-toxicity, in addition to providing bioavailability and cost-effectiveness.

### 2.3. Nanoemulsions 

**Figure 3:**
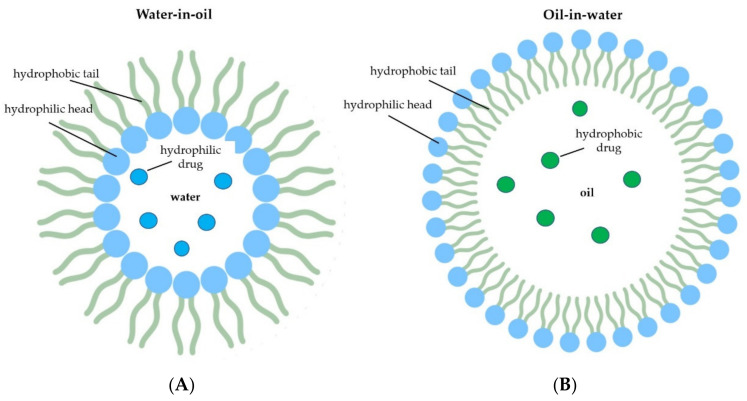
Schematic representation of nanoemulsions. Water-in-oil, where water and a hydrophilic compound are dispersed in an oily medium (**A**) and oil-in-water, in which the oil and the hydrophobic compound is dispersed in an aqueous phase (**B**).

Nanoemulsions (NEs) are a colloidal system synthetized by the dispersion of two immiscible liquids, such as oil and water, stabilized by a surfactant compound, forming droplets ranging from 20 to 100 nm. In this system, the active principle, a hydrophilic or hydrophobic molecule, is dissolved in water or oil, and the active solvent is then emulsified in water (oil-in-water) or oil (water-in-oil) in the presence of surfactants [118] (Figure 3). In the food industry, NEs have been applied to improve the solubility, stability, bioavailability, and functional property of many bioactive compounds incorporated in beverages, sauces, desserts, and dairy products, as well as for the fortification, texture enhancement, and overall product quality of these foodstuffs [118,119].

Peanut, corn, olive, and sesame oils and medium chain triglycerides (6 to 12 C) are commonly used as active principle-lipid coatings, while Tween-20, Tween-80, glycerol monooleate, soya lecithin, and polyoxyethylene can be employed as emulsifiers to stabilize the oil-water interface [120,121]. Encapsulation improves the solubilization of hydrophobic functional compounds, protecting them against oxidation and gastrointestinal tract chemical modifications, increasing their bioavailability [122]. Polyphenols are good candidates to be transported in NEs due to their good lipophilicity and their inherent stability limitations and water insolubility as well as their low bioaccessibility and bioavailability, which can be resolved by lipid encapsulation [123].

In this sense, tea polyphenols from *Camellia sinensis* (400 mg) were encapsulated in corn oil using water and polysorbate 80 (oil-in-water), generating stable 99.4 nm NEs, which display no significant size modifications, aggregation, or pH after 20 days of storage at 4, 25, or 40 °C [124]. Trans-resveratrol has also been incorporated into oil-in-water NEs, generating 241 nm nanoparticles with 99.9% efficiency encapsulation. NEs were synthesized by the interfacial deposition method, in which trans-resveratrol was incorporated into a matrix consisting of poly(e-caprolactone), triglyceride, sorbitan monostearate, and polysorbate 80. The trans-resveratrol in the NEs was stable for 90 days at room temperature, exhibiting no physical-chemical changes [125].

According to Sessa et al. [126], the trans-resveratrol encapsulated in different food-grade NEs generated by high pressure homogenization and containing peanut oil and emulsifiers soy lecithin, glycerol monooleate, and sugar ester exhibit stable diameters (150–270 nm) at 4, 30, and 55 °C after 30 days in comparison with NEs containing a synthetic emulsifier (>50 nm), demonstrate the superior technological quality conferred by naturally available food-grade compound NEs concerning shelf life. In addition, the oxidative stability of trans-resveratrol in NEs (0.01%) is greater according to increasing amounts of peanut oil (9–27.9%) and soy lecithin (1–2.1%), reaching 92% stability under storage and ultraviolet (UV) light compared with both free resveratrol and that encapsulated by the synthetic emulsifier formulation, reported as 45% and 82%, respectively.

Biocompatible NEs developed using isopropyl myristate combined to ionic and non-ionic surfactants (Tween-80, labrasol, and maisine) plus transcutol as an aqueous solvent (oil-in-water) were produced for curcumin encapsulation by emulsification at low energy. Curcumin-loaded NEs were 165 nm in size, with a low PDI of 0.072 and stable for 60 days at room temperature [127]. This indicates that NEs from food grade are good alternatives for polyphenol encapsulation due to facilitated production under mild processing conditions, resulting in high encapsulation efficiency and physicochemical stability [122,126,128]. 

### 2.4. Nanoliposomes and Niosomes

**Figure 4:**
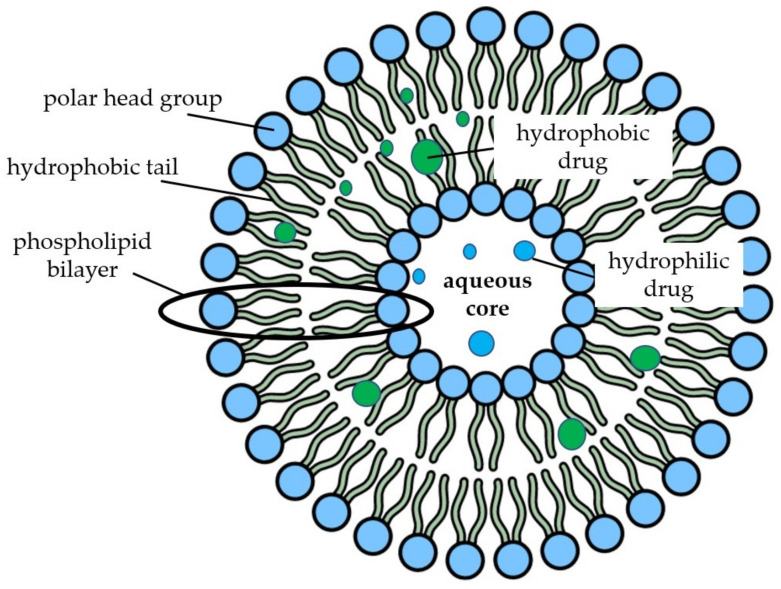
Schematic representation of nanoliposomes containing hydrophilic and hydrophobic entrapped compounds.

Liposomes are used in the agricultural, food, and pharmaceutical industries as carriers for both nucleic acids and nutraceutical products such as vitamins, proteins and enzymes, and whole herbal extracts [129]. Due to their similarity to biological membranes, liposomes are considered the standard gold model for cell membrane studies and human applications [130,131]. These spherical lipid vesicles contain homocentricly arranged phospholipid bilayers around an aqueous core with the polar heads of the phospholipids located on the outer and inner surfaces of the membranes in contact with the aqueous environment, while fatty acids form the hydrophobic core of the membranes, resulting in liposome amphiphilicity and allowing the encapsulation of hydrophilic or lipophilic agents [132,133] (Figure 4). 

The main liposome components are lipids or phospholipids that can contain sterols incorporated in their structure. Cholesterol is the most widely used sterol in liposome syntheses, as it mimics the cell membrane and increases the stability of the lipid structure by modulating the lipid bilayer fluidity [134].

In addition, niosomes, self-assembled vesicles, are formed by hydration of non-ionic surfactant, cholesterol, or other amphiphilic molecules and are able to transport both hydrophilic and hydrophobic molecules. Their chemical stability, easy preparation and low-cost production make them versatile nanocarriers for the delivery of drugs and bioactive compounds by several routes including oral, ocular, topical, pulmonary, parenteral, and transmucosal [135,136,137].

There is evidence that NLs may comprise good delivery nanodevices to supplement polyphenols in a safe and quick way to individuals presenting inflammatory and oxidative conditions [138].

Nanoliposomes (NLs) and liposomes display identical chemical structures and thermodynamic properties, but the former exhibit greater surface area, stability, solubility, bioavailability, delayed body clearance, and precision targeting [139,140]. In this context, NLs are employed to improve the stability, poor water solubility, and oral bioavailability of many polyphenols [141,142].

Mucus is the first barrier for nutrients from the oral route. Curcumin-NLs with improved stability and mucoadhesive properties were synthesized through encapsulation in L-α-phosphatidylcholine at a 1:10 (*w*/*w*) ratio with chitosan (0.1%) by the ethanol injection method. These NLs displayed 123 nm average diameter, PDI reduced to 0.2 and stability at 4 °C and 25 °C during 3 months of storage. Furthermore, due to the inherent mucoadhesiveness of chitosan, NLs formulated with this polymer exhibit increased affinity for mucin (68%), the major mucus component, when compared to NLs prepared without chitosan, as revealed in a mucin adsorption model [143]. These results were corroborated by chitosan-coated NLs possessing increased mucus affinity synthetized by Filipović-Grcić et al. [144] and Andersen et al. [145].

Liposomal quercetin encapsulation (0.02 mg·mL^−1^) in rice bran phospholipids (0.13 mg·mL^−1^) by the thin film-sonication method yielded spherical 157.33 nm NLs with an 84.9 % encapsulation efficiency. The NLs were stable at 4 °C and 27 °C for five months as indicated by the unchanged DPPH radical-scavenging assays and high quercetin NL retention (>97%). In addition, NLs displayed limited gastric degradation with a sustained release of 20% of quercetin after 4 h in gastric fluid, followed by a sustained intestinal release of 70% up to 24 h [146].

The nano-encapsulation of a mixture of bioactive substances in the same particle can be a good strategy for oral antioxidant co-supplementation with greater physicochemical potential and biological property retention [147]. Chen et al. [128] co-loaded quercetin and EGCG into NLs formulated under optimal conditions (lecithin-total polyphenol ratio of 25:1, lecithin-cholesterol ratio of 6:1, lecithin-Tween 80 ratio of 8:1, and ultrasonic time of 2 min). The mean size, PDI and zeta potential of these NLs were 111.10 nm, 0.259, and –19.83 mV, with an encapsulation efficiency of 61.7% and 64% for quercetin and EGCG, respectively. The NLs displayed a 4% size increase after 30 days at 4 °C, but no significant change in PDI and the zeta potential. The co-encapsulation of these polyphenols promoted a synergistic antioxidant effect evaluated against radical DPPH (IC50 21.7 mg·L^−1^), as evidenced by the γ value (0.91) [128].

This evidence indicates that nanoliposomes represent a promising tool for polyphenol delivery.

## 3. Nano-Encapsulated Polyphenols Bioaccessibility and Bioavailability

Polyphenols display antioxidant and anti-inflammatory effects that play a preventive and therapeutic role in chronic diseases such as cardiovascular and neurodegenerative conditions, as well as neoplasms [148]. To be effective in the human body, polyphenols must be available and present in pharmacological concentrations to target organs or tissues after intestinal absorption. When ingested, polyphenols that comprise part of food matrices suffer a series of chemical modifications, being hydrolyzed and conjugated by methylation, alkylation, sulfation, and glucuronidation reactions in the small intestine and liver during absorption and before reaching the systemic circulation. The remaining compounds reach the large intestine and colon, where they may undergo further metabolization by the microbiome to generate other functional compounds or be excreted in the feces and urine [149]. Conversely, the interaction of polyphenols with food matrix constituents, such as proteins, fibers, and minerals can negatively impact their bioaccessibility and oral bioavailability [150,151,152,153,154].

As mentioned previously, the technological functionalities of NPs regarding polyphenols solubility, release kinetics, and chemical structure protection when exposed to different physicochemical conditions are well recognized. However, in vitro assays should also be performed to address the stability of nano-encapsulated phenolics under unfriendly gastrointestinal conditions. The oral bioavailability of nano-encapsulated phenolics must be evaluated in pre-clinical and clinical assays to reinforce and prove the advantage of nano-encapsulation concerning bioaccessibility and bioavailability gains, since they represent restrictions toward the effectiveness of polyphenols as supplements for well-being or as an adjuvant therapy to treat chronic diseases. Several reports regarding phenolic compound gastrointestinal stability, longer residence times, and increased oral bioavailability after nano-encapsulation, with no cell or tissue damage, are available in the literature.

The trans-resveratrol-loaded into lipid core polymeric nano-capsules formed by poly(ε-caprolactone) and capric/caprylic-triglycerides allowed for increased accumulation of this antioxidant in the liver, kidney, and brain tissues of healthy rats compared to those supplemented with free resveratrol following intraperitoneal or gavage administration of 5 mg·kg^−1^ for 14 days. The trans-resveratrol treatment may have provoked the development of gastrointestinal injuries due to inhibition of cyclooxygenase-1, while animals treated with trans-resveratrol nano-capsules exhibited lower lesion rates in the duodenum, jejunum, and ileum compared with free-resveratrol intake. It seems that an improvement in the safety usage of this antioxidant regarding gastrointestinal trait preservation can be achieved when this antioxidant is included in NPs [125].

Peanut oil and soy lecithin (oil in water) were used to encapsulate resveratrol and evaluate a simulated gastric and intestinal digestion, which resulted in resistance indicating that NEs allow full resveratrol availability for intestinal absorption. The size of the NEs of 250 nm enabled them to cross Caco-2 cell membranes and exert their intracellular antioxidant activity against hydrogen peroxide [126].

The same simulated gastrointestinal fluid resistance is noted for casein-NPs. Resveratrol-casein-NPs exhibit a 10-fold higher resveratrol bioavailability and displayed stable plasma levels for 8 h after a single 15 mg·kg^−1^ oral administration in Wistar rats compared with a 4 h plasma level maintenance observed for free resveratrol. A good correlation between in vitro bioaccessibility and the resveratrol fraction absorbed in vivo (R^2^ = 0.99) indicates that NPs are able to carry the resveratrol to the intestinal epithelium and promote a higher absorption rate of this polyphenol [71].

Casein-hydroxypropyl-β-cyclodextrin NPs loaded with quercetin have been reported as resistant to gastrointestinal pH conditions and allow for the controlled release of 20% of quercetin in gastric fluid (pH 1.2), and 60% after 4 h in intestinal fluid (pH 6.8), while free quercetin is fully released when reaching gastric conditions. In rats, the plasma levels of nano-encapsulated polyphenols were prolonged for 72 h and 37% of bioavailability was reported after the oral ingestion of 25 mg·kg^−1^, as demonstrated by a higher plasma half-life, mean residence time, and area under the curve (AUC), while free quercetin resulted in elevated plasma levels for only 8 h with a 4% bioavailability [155].

Ex vivo passive paracellular and transcellular transport models indicate that chitosan-TPP NPs enhance polyphenol absorption and transport across excised mouse jejunum in Ussing chambers. Catechin (3.6 mg·mL^−1^) and epigallocatechin (6.2 mg·mL^−1^) in the NPs were significantly better transported through jejunum tissue compared to non-encapsulated polyphenols (302.1 vs 206.8 ng·cm^−2^ and 102.7 vs 57.9 ng·cm^−2^, respectively). This bioavailability improvement was attributed to the stability afforded by NPs, resulting in higher available polyphenol concentrations to flux across the tissue [113].

In a pharmacokinetic assay, a single 100 mg·kg^−1^ dose of chlorogenic acid entrapped in chitosan-TPP NPs (210 nm) was administered to Wistar rats, leading to a longer polyphenol residence time in plasma, extended time to reach maximum plasma concentration, and a greater area under the curve (*p* > 0.01), configuring a slow and prolonged chlorogenic acid release in plasma and superior oral bioavailability [105].

In another study, Wistar rats received an oral supplementation of 50 mg·kg^−1^ of resveratrol encapsulated in carboxymethyl-chitosan NPs. The resveratrol-NPs offered in a single dose with an absorption time of 1.76 h, reaching a plasma peak after 6.83 h, and excreted in 20.5 h, while free resveratrol was rapidly absorbed at 1.12 h, with a plasma peak at 3.21 h, and excretion at 5.44 h. Resveratrol nano-encapsulation in carboxymethyl-chitosan NPs thus ensured a longer permanence of the antioxidant in the systemic environment, resulting in a substantial 3.5-fold increase in bioavailability [110].

Distearoyl phosphatidyl choline and cholesterol liposomal formulations were used to load resveratrol (10 mg), improving gastrointestinal resistance against destabilization by bile salt and sodium taurocholate solutions. In addition, rats supplemented by resveratrol-NLs at 50 mg·kg^−1^ exhibited sustainable resveratrol release in plasma with a two-fold increased bioavailability and a maximum plasma resveratrol concentration of 0.42 μg·mL^−1^ and AUC 1.44 µg·h·mL^−1^ after 6 h, while free resveratrol was found at 0.33 μg·mL^−1^ at 30 min with an AUC of 0.633 µg·h·mL^−1^. In the same assay, nano-encapsulated resveratrol was found to be predominantly absorbed as the unmetabolized chemical structure (RSV-3-O-glucuronide), while free-resveratrol intake generated higher plasmatic metabolite levels [156], corroborating greater stability and bioavailability conferred by NLs.

In short, poor polyphenol bioaccessibility and bioavailability can be overcome by their encapsulation in NPs or NEs obtained from natural food-grade polymers. Such benefits are the result of the chemical protection and enhanced solubility conferred by the polymeric matrices involving polyphenols, guaranteeing a greater number of intact polyphenol molecules able to reach absorption sites. In addition, the nanometric size of the particles, cell adhesion, and permeation properties facilitate polyphenol diffusion through membranes, increasing their absorption through the intestinal epithelium and ensuring bioavailability. Finally, the residence time of NPs containing polyphenols in biological compartments also increases their bioactive performance.

## 4. Critical Analysis of the Effectiveness of Nano-Encapsulated Polyphenols on Cardiovascular Performance: Pre-Clinical and Clinical Trials

The protective role that polyphenols play in pathophysiological disorders that lead to endothelial dysfunctions and cardiovascular events is strongly supported by scientific evidence. Polyphenols are pleiotropic compounds that exert epigenetic, antioxidant, and anti-inflammatory effects on various tissues, including vascular and cardiac tissues [157,158,159,160]. In humans, the regular intake of polyphenols such as curcumin, catechin, quercetin, anthocyanins, and resveratrol, attenuate hypertension, hyperglycemia, hyperlipidemias, oxidative stress and overall inflammatory status, which are the major physiological conditions able to trigger cardiovascular events [8,9,10,22,160,161].

However, attempts to demonstrate the effect of isolated antioxidant molecules following oral intake on antioxidant status, lipid peroxidation, and inflammatory markers in humans have failed, perhaps due to the chemical instability of these molecules along the gastrointestinal trait alongside their low bioavailability in biological fluids [162,163,164,165].

In general, clinical trials designed to evaluate the effects of polyphenol-loaded NPs on cardiovascular performance are scarce. Boarescu et al. [166,167,168] approached this knowledge gap by evaluating the effects of curcumin nano-encapsulated on cardiac tissue in animal model, reporting that curcumin-NPs protect against damage to cardiomyocytes, acute myocardial infarction, and post-infarction cardiac injury induced by isoprenol in healthy and diabetic rats. The effects of NPs at 200 mg·kg^−1^ were superior to non-encapsulated curcumin and governed by inherent NP properties, which increase polyphenol bioavailability and delivery to the infarcted area, while also affecting pro-inflammatory cytokines and oxidative stress, as expected for curcumin, albeit enhanced by nano-encapsulation [169]. In addition, electrocardiograms and blood pressure tests have reinforced the beneficial role of nano-encapsulated curcumin on cardiac function. Although the nano-encapsulating polymer was not specified, the aforementioned measures indicate a promising therapeutic efficacy of nano-encapsulated polyphenols. Ray et al. [170] also demonstrated that curcumin loaded in carboxymethyl chitosan-NPs and offered at an oral dose of 5 mg·kg^−1^ was successfully delivered to compromised myocardium, reducing cardiac hypertrophy and apoptosis in male Wistar rats.

Resveratrol-loaded zein-NPs were able to reduce serum tumor necrosis factor (TNF-α) levels in 15% compared to free resveratrol after the orally administration of 15 mg·kg^−1^ doses for 7 days to C57BL/6J female mice [171]. As TNF-α is one of the most potent pro-inflammatory cytokines involved in a broad range of immune and inflammatory processes such as atherosclerosis, the cardiovascular effect of nano-encapsulated resveratrol should be considered as a cardioprotective dietary intervention [172]. The same effect was observed after the administration of quercetin-loaded in zein-2-hydroxypropyl-β-cyclodextrin NPs at 25 mg·kg^−1^ for 1 week, which resulted in a significant TNF-α decrease when comparing animals treated with quercetin and the control group (*p* < 0.05 and *p* < 0.01, respectively) [173].

Anti-inflammatory effects were also demonstrated when cherry extracts (*Prunus avium* L.), which are rich in polyphenols (quercetin and cyanidin-3-glucoside), were encapsulated in quaternary ammonium chitosan-NPs. NPs displaying 2 µg·m^−1^ of gallic acid equivalents (GAE) were incubated with human endothelial cells after lipopolysaccharide-induced inflammation, significantly reducing TNF-α, interleukin-6 (IL-6), NO, and cyclooxygenase-2-dependent (COX-2) prostaglandin E2 levels and increasing anti-inflammatory IL-10 [174].

Curcumin in micelle-NPs significantly reduced the mRNA expression and secretion of serum IL-1β and IL-6 cytokines in COVID-19 patients supplemented for 14 days with 160 mg of NPs [175]. COVID-19 is a high-incidence disease displaying high mortality rates worldwide, characterized by the increased secretion of pro-inflammatory cytokines and acute inflammation that compromises the cardiovascular system [176].

As mentioned previously, polyphenols can protect macromolecules and tissues against oxidative damage due to their antioxidant properties, which can be enhanced by nano-encapsulation. Oxidative stress is a physiopathological condition enrolled in risk factors for cardiovascular diseases, and the monitoring of end products generated from the oxidation of biological macromolecules in biological fluids as well as intracellular antioxidant enzyme levels are used to assess the degree of cell damage and the therapeutic potential of drugs and bioactive compounds [177]. Oxidative stress markers such as NOx, malondialdehyde (MDA), total oxidative stress, catalase, and total antioxidant capacity, were normalized in the plasma of Wistar rats following the intake of a liposomal curcumin formulation based on dipalmitoyl-sn-glycero-3-phosphocholine, 1,2-distearoyl-sn-glycero-3-phosphoethanolamine-N-[amino(polyethylene glycol)-2000] (PEG-2000-DSPE), and cholesterol (10 and 20 mg·kg^−1^) for 8 days after oxidative stress development induced by gentamicin. Concerning all the evaluated oxidative damage indicators, curcumin in NLs was more effective than free curcumin supplementation [178].

Oxidized amino acids (free or protein-bound) are typical oxidative stress markers, and dityrosine bonds formed in low density lipoprotein (LDL) particles comprise atherogenesis indicators [179]. Protein oxidation and consequent cross-linked dityrosine formation are inhibited by quercetin and myricetin individually loaded in chitosan-NPs. Both polyphenols inhibited ribonuclease A oxidation and the crosslink induced by potassium persulfate (K_2_S_2_O_8_) at 25 µg·mL^−1^ [109].

Resveratrol is known to protect the cardiovascular system by alleviating oxidative stress and controlling blood glucose [180]. Synthesized PEG-cholesterol NLs (200 nm) containing resveratrol (2.5–100 µg·mL^−1^), displayed significant glycemic and insulinemic control in ß TC cells following diabetes induced by glucose and streptozotocin. In addition, cellular oxidative stress caused by long exposure periods to glucose and streptozotocin was mitigated following 24 h incubation with resveratrol-NLs, which significantly restored superoxide dismutase (SOD) and glutathione peroxidase (GSH-Px) levels compared to in-solution resveratrol administration [181].

Anthocyanins, the major polyphenol pigment in plants, can also reduce physiopathological damages associated with type 2 diabetes, but with limited effects due to poor chemical stability, aqueous solubility, and bioavailability [182,183]. Anthocyanin-loaded niosomes, administered as a functional drink, may result in beneficial effects on the reversal of metabolic abnormalities associated with diabetes and obesity in mice fed a fat diet. Niosomes containing anthocyanins offered to obese and insulin-resistant mice at 160 µg·mL^−1^ over a four-week period improved glucose tolerance and insulin sensitivity and led to a significant reduction in fasting glucose, insulin levels, and leptin. Furthermore, decreases in blood insulin levels of animals treated with anthocyanins-loaded niosomes were more effective than free-anthocyanin and as effective as metformin, demonstrating that niosomes may increase polyphenol carbohydrate metabolism effectiveness [184].

Quercetin loaded into alginate-chitosan NPs (10 µg·mL^−1^) displayed protective effects against peroxidation induced by iron/ascorbic acid in isolated rat liver microsomes, as determined by significant MDA level decreases. In addition, quercetin NPs containing a higher chitosan concentration (3 mg·mL^−1^) resulted in significant protection against t-BuOOH-induced GSH depletion, increasing the levels of this tripeptide by 183% [77]. In short, antioxidants, especially those belonging to the polyphenol class, which represent the most abundant dietary bioactive compounds, are arguably excessive reactive oxygen and nitrogen species removers, also able to regulate endogenous defenses and attenuate inflammation, which are key processes in the progression of cardiovascular diseases when in physiological imbalance. However, food processing, environmental factors, and gastrointestinal and the systemic instability of the human body limit polyphenol bioavailability and bioactivity, compromising their oral administration effectiveness. A large number of in vitro assays, cell cultures, and animals comprising storage, digestive, pharmacokinetics, absorptive, and adverse metabolic models, have demonstrated that the application of nanotechnology in polyphenol encapsulation indicates their use as a therapeutic agent for humans.

The current scientific framework demonstrates that the synthesis of nano-encapsulated polyphenols loaded in food grade polymers and lipids can be physiologically safe. NPs increase environmental and digestive polyphenol stability, maintaining these molecules intact during the enteric route to intestinal absorption sites. After becoming more accessible to the intestinal epithelium, nano-encapsulated polyphenols exhibit greater mucoadhesiveness and display the ability to be taken up in greater amounts by intestinal cells, reaching systemic levels in their non-metabolized form. The high concentrations of intact nano-encapsulated polyphenols that cross the intestinal epithelium allows them to reach high systemic levels while, at the same time, be released gradually, with a longer half-life and residence time compared to non-encapsulated polyphenols. The gradual release and long permanence of polyphenols in their active form in the human body conferred by NPs thus allow for their effective use as an adjuvant therapy against cardiovascular diseases.

## 5. Nanoparticles: Safety and Cytotoxicity

The application of nanotechnology has increased substantially in science and in the materials technology, medicine, food, and agriculture fields. Several commercially available products, such as toothpaste, wound dressings, sunscreens, and skin creams contain NPs in their formulations, mostly produced using silver, zinc oxides, silica, and gold [185,186,187]. This represents increasing human exposure to NPs, which can be inhaled, ingested, and absorbed transdermally, stimulating biosafety questions. The safety of NPs is questioned due to their physicochemical characteristics (size, shape, charge, etc.), which makes them easier to permeate, penetrate, and be retained in cells and tissues, which may or may not be desirable, as NPs can accumulate in organs for considerably long periods of time without adequate clearance, culminating in oxidative tissue stress, inflammation, and cell death. In blood, NPs display the ability to adsorb proteins from the physiological fluid and inducing platelet aggregation, coagulation, and hemolysis [188,189]. In addition, information concerning NP behavior in the body is not fully elucidated, compromising knowledge on their harmful and beneficial effects [190]. In this sense, the European Food Safety Authority (EFSA) [191] has stablished guidelines on the physical-chemical characterization and analytical methods used in determining the systemic behavior of nanoproducts, which includes absorption, metabolism, cell-NP interaction, excretion, and toxicity mechanisms. Although this document does not represent an official regulatory guideline for nanotechnology, no other regimentations are available. Other agencies, such as the European Medicine Agency (EMA), Organization for Economic Co-operation and Development (OECD), and National Nanotechnology Initiative (NNI), have established provisional guidelines in an attempt to partially regulate nanotechnology application standards [192].

In contrast, the use of food-grade NPs, mainly in the medicine and food technology fields, represents a better solution in view of the uncertainty concerning nanoproduct safety. Food-grade polymers are biodegradable, do not generate toxic by-products, are biocompatible, non-immunogenic, and permeable to biological barriers and certain polymers display biological activities. In addition, most are classified as “substances generally recognized as safe” (GRAS) status designated by the Food and Drug Administration (FDA) [30,193]. These characteristics have stimulated the use of compounds obtained from natural sources, such as polysaccharides, proteins and lipids, as raw materials to build nano-capsules for the delivery of therapeutic agents and other nanotechnological purposes with no adverse human health effects [194]. The number of nano products based on food-grade polymers submitted to the FDA over the last two decades has increased, intended predominantly for intravenous and oral delivery. Liposomes were the first FDA-approved drug nanocarrier and are included in one of the most requested and approved nanoproduct category (33%), followed by nanocrystals (23%), emulsions (14%), iron–polymer complexes (9%), and micelles (6%), while the last 14% of requests consist mainly of nanoproducts coated by polymers, or drug-protein complexes [195,196].

Currently, a number of food-grade NP coated drugs for use in medicine, such as Abraxane^®^, Doxil^®^, and Zinostatin/stimalmar^®^, among others, are approved for commercialization [197]. To the best of our knowledge, curcumin-NPs, known as THERACURMIN and provided by Theravalues Corporation (Tokyo, Japan), are the only food-grade polyphenol coated with a biodegradable material commercially available as a non-drug marketed for dietary supplementation. These NPs are 190 nm in size and consist of 10% curcumin, 2% other curcuminoids such as demethoxycurcumin and bisdemethoxycurcumin, 46% glycerin, 4% gum ghatti, and 38% water.

Other formulations containing encapsulated curcumin in solid lipid NPs (WO/2007/103435), oil emulsion NPs (CN1736369), and chitosan NPs (WO2010013224A2) have been formulated and patented for human applications. Several studies have demonstrated that curcumin may be beneficial in different disorders, such as Alzheimer’s disease, cardiovascular diseases, and psoriasis, due to its anti-inflammatory, antioxidant, and anti-cancer effects [198,199]. For this reason, curcumin has often been administered in several unhealthy conditions [200].

In vitro cytotoxicity assays are performed as part of product characterization during the formulation of NPs from natural polymers, in order to assess or rule out potential cell damage that can make their use unfeasible. Although pre-clinical and clinical trials are more appropriate, they are expensive and slower. In addition, pre-clinical trials in animal models have been discouraged due to animal sacrifice, as recommended by the European Commission, which guides the scientific community on the replacement of animal trials based on the “replacement, reduction, and refinement of animal use” principle [201]. 

Cultures comprising Caco-2 intestinal cells, erythrocytes, respiratory tract cells, epithelial cells, hepatocytes, and embryonic cells are of the in vitro models applied in cytotoxicity food-grade NPs coated polyphenol assays (Table 1).

Das et al. [86] reported no cytotoxicity of sodium alginate-chitosan NPs loaded with curcumin after absorption by HeLa cells. According to a test conducted by [75], starch NPs loaded with catechin, epicatechin, epicatechin-3-gallate, and proanthocyanins exhibited good compatibility and no toxicity to fibroblast embryonic cells. No cytotoxic effect was found in healthy lung cells after exposure to insulin-loaded chitosan-dextran NPs [99]. Finally, in vivo toxicological evaluations were performed after a 14-day oral administration of quercetin loaded in chitosan-alginate NPs to rats (0.5 mL·100 g^−1^) [77]. No mortality or altered animal behavior was observed. The relative mass of the liver, histological sections, liver function, and hematological parameters were not altered due to NP intake, indicating no health risks and suggesting the possibility of safe oral intake.

However, tests on healthy cells are scarce due to the emphasis placed on the effects of NPs on tumor cells in different tissues, i.e., gastrointestinal and respiratory tracts and the brain [52,62,65,87,204].

Although they do not clarify the mechanisms of toxicity or cell death, the colorimetric methods thiazolyl blue tetrazolium bromide (MTT) and (3-(4,5-dimethylthiazol-2-yl)-5-(3-carboxymethoxyphenyl)-2-(4-sulfophenyl)-2H-tetrazolium) (MTS) concerning mitochondrial function and lactate dehydrogenase (LDH) regarding cell membrane integrity are the most applied techniques to assess NP cytotoxicity [205]. Results obtained by in vitro tests should be interpreted cautiously, due to their limitations and the fact that NPs behavior in physiological media can be affected by several factors that may influence cytotoxicity and that are not considered by in vitro tests [206].

Despite the scarcity of clinical trials with NPs, their oral and topical use in humans is well tolerated, with no severe side effects or toxicity in administration periods ranging from 4 to 12 weeks [31,207,208]. Recently, two lipid-NPs vaccines prepared using an ionizable lipid, phospholipid, cholesterol PEGylated lipid and distearoylphosphatidylcholine, namely BNT162b2 (Pfizer) and mRNA-1273 (Moderna), were applied to over 60 thousand volunteers (>16 yo) in randomized and placebo-controlled clinical trials concerning their safety and effectiveness against severe acute respiratory syndrome coronavirus 2 (SARS-CoV-2), after a two-injection regimen. These vaccines have been shown to be safe in animals and humans, with a low incidence of adverse effects in volunteers and an efficacy greater than 90% in preventing severe COVID-19 [209,210,211,212]. This evidence reinforces the possible safety of bioactive and nano-encapsulated therapeutic agents into food-grade polymers and indicates that their beneficial roles overlap with possible harmful human health effects.

## 6. Conclusions

The biological effects of polyphenols on cardiovascular health are irrefutable, although their effectiveness in clinical trials in humans may be compromised due to the physiological variabilities among individuals that can compromise polyphenol physical-chemical integrity. However, the major challenge is to delivery these bioactive molecules through distinct routes i.e., oral, parenteral, or topical, to fulfill therapeutic needs. Polyphenol effectiveness depends on antioxidant characteristics, which requires that their chemical structure be preserved along the gastrointestinal tract during intestinal absorption, when in the bloodstream, when reaching the target cell these compounds, and when crossing plasmatic or intracellular membranes in order to exert their bioactivity. The incorporation of polyphenols into NPs may keep these compounds protected from hydrolytic enzymes, oxidation, and other stringent physicochemical conditions throughout the gastrointestinal and systemic pathways, and, at the same time, overcome polyphenol bioaccessibility and bioavailability. Distinct, efficient, and safe nanocarriers have been described and seem to be able to deliver polyphenols in an adequate kinetic release guaranteeing a sustained pharmacological concentration of polyphenols for several hours. Pre-clinical assays have demonstrated that nano-encapsulated polyphenols such as ferulic acid, quercetin, gallic acid, resveratrol, catechin, epicatechin, epigallocatechin, proanthocyanidin, and curcumin, as well as whole extracts from grape seeds and cherries, exhibit no toxicity to human cell lineages. The use of polyphenols in NPs should be encouraged for the incorporation and fortification of foods and beverages, in addition to their application in the manufacturing of nutraceuticals in order to conveniently assist in the prevention of and in adjuvant therapies against multifactorial and physiopathological complex conditions, such as cardiovascular diseases and other chronic health impairments.

Abbreviations: ABTS, 2,2′-azino-bis(3-ethylbenzothiazoline-6-sulfonic acid; ADMA, asymmetric dimethylarginine; AUC, area under the curve; BSA, bovine serum albumin; CHC, chitosan chloridate; CMC, carboxymethyl chitosan; COX, cyclooxygenase; DDAH, dimethylarginine dimethylamino hydrolase; DPPH, 2,2-diphenyl-1-picrylhydrazyl; EFSA, European Food Safety Authority; EGCG, epigallocatechin gallate; EMA, European Medicine Agency; eNOS, nitric oxide synthase; FDA, Food and Drug Administration; GRAS, generally recognized as safe; GSH-Px, glutathione peroxidase; HSA, serum albumin; IL, interleukin; K_2_S_2_O_8_, potassium persulfate; LDH, lactate dehydrogenase; LDL, low density lipoprotein; MDA, malondialdehyde; MTS, (3-(4,5-dimethylthiazol-2-yl)-5-(3-carboxymethoxyphenyl)-2-(4-sulfophenyl)-2H-tetrazolium; MTT, thiazolyl blue tetrazolium bromide; mV, millivolt; NEs, nanoemulsions; NF-κB, factor nuclear kappa B; NLs, nanoliposomes; NNI, National Nanotechnology Initiative; NO, nitric oxide; Nrf2-ARE, nuclear factor erythroid-2-related factor—antioxidant response element; NPs, nanoparticles; OECD, Organization for Economic Co-operation and Development; PEG, Polyethylene glycol; PDI, polydispersity index; PPAR-γ, peroxisome proliferator-activated gamma receptor; ROS, reactive oxygen species; SOD, superoxide dismutase;. TNF-α, tumor necrosis factor; TPP, sodium tripolyphosphate; UV, ultraviolet.

## Figures and Tables

**Table 1:** Cytotoxicity testing of food-grade NPs in healthy cultured cells.

Wall Polymer	Polyphenol	Cytotoxicity Assay	Cell Culture	Exposure Time	NP Concentration	Cell Viability	Reference
Thiolated quaternary ammonium-chitosan	Grape seed polyphenols	WST-1	EPCs	3 h	0–4.9 μg·mL^−1^	100%	[202]
Soy protein nanoemulsion	Catechins	MTT	Caco-2	24 h	75 and 500 μg·mL^−1^	95 and 85%	[203]
Starch NPs	Catechin, epicatechin, epigallocatechin, proanthocyanidin, individually encapsulated and assayed	MTT	MEF	24 h	0.015–0.250 μg·mL^−1^	>80%	[75]
Transcutol-based nanoemulsion	Curcumin	MTT	nasal epithelium	72 h	0.012–1 mg·mL^−1^	100%	[127]
Chitosan-alginate NPs	Quercetin	MTT LDH	HepG2	24 h	10 μg·mL^−1^	100%	[77]
Zein-casein-lysine NPs	Ferulic acid	MTT	Caco-2	24 h	0.1–1000 μg·mL^−1^	94%	[72]
Zein-casein-lysine NPs	Ferulic acid	MTT	Caco-2	4 h	100 and 300 μg·mL^−1^	100%	[72]
Liposomes NPs	Resveratrol	MTT	ß-pancreatic	24 h	25 μg·mL^−1^	>90%	[181]
Quaternary ammonium chitosan NPs	Cherry extract	WST-1	HUVEC	2 h	2 μg·mL^−1^ GAE	77%	[174]
liposome-phosphatidylcholine from soybean	Quercetin and gallic acid alone and co-loaded	CC8-K	RAW 264.7	24 h	1, 10 and 50 μg·mL^−1^	100%	[138]

CC8-K: Cell counting Kit-8; EPCs: Endothelial progenitor cells; GAE: Gallic acid equivalents; HaCaT: Human keratinocyte cells; HepG2: Hepatocellular carcinoma cells; HUVEC: Human umbilical vein endothelial cells; LDH: Lactate dehydrogenase; MEF: Mouse embryonic fibroblast; MTT: Thiazolyl blue tetrazolium bromide; WST-1: Water-Soluble tetrazolium salt.
